# The cellular and humoral immunity assay in patients with complicated urolithiasis

**Published:** 2017

**Authors:** E Ceban, P Banov, A Galescu, D Tanase

**Affiliations:** * Department of Urology and Surgical Nephrology, “Nicolae Testemiţanu” State University of Medicine and Pharmacy, Chişinău, Republic of Moldova; ** Clinic of Urology, Clinical Republican Hospital, Chişinău, Republic of Moldova

**Keywords:** complicated urolithiasis, immunological evaluation, cellular and humoral immunity assay

## Abstract

Especially complicated, renal lithiasis contributes to the general inflammatory syndrome development that interferes with nonspecific, humoral and cellular immune system. The surgical treatment of nephrolithiasis is closely related to drug therapy of urinary infection, one of the reasons being the reduction of the immune status. The work is performed by evaluating the immunological status preoperatively in 58 patients with ***complicated*** lithiasis. The analysis of the status in these patients demonstrated that complicated urolithiasis results in significant changes in the immune system, these changes being expressed at the cellular and humoral level of immunity.

## Introduction

Renal lithiasis (LR) is a complex syndrome which includes: impaired metabolism of a number of lithogenic substances, their transportation disturbance through the intestine, kidneys and urinary tracts, pathological changes in the physicochemical and biological urine, the creation of some favorable conditions for the formation of kidneys calculi and crystals, in the human body [**1**,**2**]. This disease is a major problem in modern urology because it occupies one of the leading places in the structure of urological diseases in all the regions of the globe.

Renal lithiasis is one of the most common pathologies of the urological diseases structure and is detected in approximately 1-3% of the general population [**2**-**4**]. It is well known that urolithiasis affects more the people of productive age, being very rare in the elderly and children, having a frequency of over 70% in patients of 20-50 years, a cause that leads to a loss of working capacity [**3**,**4**]. According to the data of some authors [**5**], 8.9% of the men and 3.2% of the women suffer from urolithiasis during life.

Currently, in highly developed countries, 400 000 people from 10 million suffer from RL. Annually, new cases of RL 85000 are recorded, of which 62000 are recurrent diseases [**1**,**3**,**5**]. Global prevalence is estimated between 1-5%, 2-13% in developed countries (with a very large variation from country to country) in the developing 0.5-1%. The overall probability of the population for developing calculi varies in different parts of the world: 1-5% in Asia, 5-9% in Europe, 13% in North America. The annual incidence of urolithiasis is of about 0.1-0.4% of the population (Romania, Republic of Moldova).

Most researchers point out that, even after the first episode of sporadic migration of calculus from the kidney, there is a probability of pathology recurrence in the next five years, ranging between 27% and 50% [**10**].

RL holds the third place in the death structure causes in patients with urological diseases [**4**,**5**]. In 28.4% nephrectomy cases, these are caused by complicated RL that, if bilateral, may worsen and lead to obstructive complications and acute or chronic renal failure [**5**]. The number of nephrectomies has recently increased because of complicated and infected lithiasis in the Republic of Moldova.

There are multiple scientific publications showing that the urinary infection is an important etiologic factor of RL. An etiologic and pathogenic role in the RL development has been to a large extent, chronic pyelonephritis, but as a complication, RL can also occur in the chronic inflammation of the upper and lower urinary tracts [**3**-**7**].

In some cases, the urinary infection precedes the RL development and can serve as a trigger for its development. In other cases, it is associated with the RL arising from other infectious causes. Usually, renal calculi form with a mixed chemical composition containing phosphate at the association of the infectious and metabolic factors. In general, the urinary tract infection is detected in 80% of the RL cases [**7**].

The role of certain microorganisms in the RL development is ambiguous. A number of mycobacteria are purely a “local” cause of secondary phosphates calculi. It includes microflora that synthesizes the urea: Proteus spp., Pseudomonas aeruginosa, Enterobacter. Other micro-organisms, such as Staphylococcus, Streptococcus, Enterococcus, E coli, Klebsiella, do not eliminate urea, but can potentiate RL due to the inflammatory process itself, to the increase of mucoprotein level, urostasis, the disorders of renal lymphatic reflux and the transport of lithogenic substances in the renal tubular system. Microorganisms that do not eliminate urea do not influence the chemical composition of urinary calculi, but accelerate calculi formation of any origin on account of pathological changes in the kidneys and urine listed above [**1**,**4**,**5**,**7**].

Lately, it has been demonstrated that the calculi of the calcium oxalate chemical structure may have an infectious cause in its genesis. The intracellular non bacteria that are detected in the urinary system calculi are capable of a coat forming and producing a phosphate chemical structure which later turns into a crystallization nucleus with a subsequent particle deposition and calculi growth.

The stone formation in the kidney tissue leads directly to significant disturbances of the urodynamics in the potassium basin system with a negative effect on the urinary tracts epithelium and as a result becomes one of the important factors in triggering chronic infectious inflammatory processes in kidneys. Between the pathologies that trigger chronic renal insufficiency, secondary pyelonephritis, which develops due to the presence of calculi in the kidneys, occupies a leading position [**3**,**5**,**10**].

A very important role in stone formation as an etiopathogenetic point pertains to chronic pyelonephritis [**1**,**5**,**7**,**8**]. In the presence of calculi, 92% of the cases (100% coraliform lithiasis) result in permanent attacks of pyelonephritis.

The specialty studies showed that permanent inflammatory processes are present in the so-called aseptic lithiasis [**1**,**2**]. The urolithiasis common pathogenetic link is represented by congenital or acquired tubulopathies. A particularly important role in the recurrent kidney calculi etiopathogeny pertains to chronic pyelonephritis, being shown that the secretion acceleration of lithogenic substances depends on the activity of the inflammatory process [**1**,**2**].

The contemporary researches demonstrated that immunological mechanisms are some of the important factors in the pathogenesis of chronic calculus pyelonephritis [**7**].

Current researches have failed to fully demonstrate the pathogenic links of immune system arising at different stages of pathogenesis of urolithiasis and chronic pyelonephritis. A particular attention is paid to the immunopathology reactions and their correction [**10**].

The correction of immunopathologic manifestations in patients with complicated urolithiasis, with secondary chronic pyelonephritis, represents one of the treatment perspectives in uro-nephrology.

It is known that the long lasting microbial infectious process leads to serious disturbances in the immune status, which can then result in the development of local immune deficit or in the body’s awareness to inflammatory toxins, with the development of generalized inflammatory reactions. 

Especially complicated, renal lithiasis contributes to the general inflammatory syndrome development that interferes with nonspecific, humoral and cellular immune system. These changes are associated with an increased concentration of biologically active proinflammatory substances, that affect the functioning of various organs and systems, and locally contribute to the development and progression of fibrotic process in the renal parenchyma and potassium-basin system, affecting the urine anti-lithogenic properties [**11**,**12**]. 

In this context, it is worth mentioning that any inflammatory process is virtually always accompanied by T lymphocytes decrease. This is observed in inflammatory processes of various etiologies, without any exceptions: infections, nonspecific inflammatory processes, destructive processes of tissues and cells after surgery, trauma, combustions, infarctions, malignant tumor formations destructive processes, trophic changes, etc. Thus, the movement of the T cells is directly proportional to the intensity of the inflammatory process. To diagnose the inflammatory process itself, that has a paramount significance in the decrease in the number of the T lymphocytes in the blood. T lymphocytes react the fastest in the presence of an inflammatory process by decreasing [**11**,**12**].

A definite importance is the immunoglobulin composition assessment for the determination of predominant impairment regions (conjunctival mucosa or deeper tissues). Mucosa inflammatory processes often result predominantly in increasing the amount of IgA or in cases of lowering the body’s resistance, in decreasing the production of IgA. In the inflammation process related to the initial contact of the body with this type of antigen, in early terms, the IgM content increases and then increases the content of IgG. The raising of the level of IgG and IgA occurs at the repeated contact with this type of antigen at the primary stages of development of the inflammatory reaction.

During the therapeutic involvement with immunomodulatory preparations we should take into account that the immunomodulatory preparations interact at different pathogenic stages of immunity and certain immunomodulatory preparations used chaotically without precise instructions can give the effect of immunosuppression [**1**,**9**-**11**].

Significant impairments in renal parenchyma in inflammatory processes prove the researches and the immune status correction in patients with urolithiasis and secondary pyelonephritis.

The purpose of the research was to study the humoral and cellular immunity in patients with complicated urolithiasis to improve the treatment outcomes postoperatively and the recurrent one.

## Material and methods

The trial was conducted on a group of patients with complex renal lithiasis treated in the Clinic of Urology and Surgical Nephrology SUMPh, “Nicolae Testemitanu” Clinical Republican Hospital during the years 2010 and 2014. 

The study of inflammatory changes in patients with renal lithiasis included 58 patients with various forms of urolithiasis surgically treated and 30 people in the control group. Both groups were homogenous by gender and age. People in the control group were actually healthy. Thus, the organization of the study allowed the highlighting of specific pathological changes for complicated urolithiasis that required surgery, compared to healthy people. 

Special laboratory methods were used for the examination of cellular and humoral immunity, severity of the inflammatory process. 

***Assessment of immunity*** in patients with complicated urolithiasis was performed on admission before the treatment. The classical method of forming rosettes with sheep erythrocytes was applied to assess the changes in cellular immunity. The following parameters were assessed: active T-lymphocytes, total-T, T-morua, T-helper (Th), T-suppressor (Ts), index Th/ Ts, B lymphocytes. Humoral immunity was assessed by the quantitative determination of IgG, IgA, and IgM by the method of gel immunodiffusion (Mancini). Additionally, the level of circulating immune complexes (CIC) was determined spectrophotometrically. The study was conducted in the immunological laboratory of the Clinical Republican Hospital. 

***General inflammatory status*** was assessed by examining the level of cytokines and proinflammatory biologically active substances: interleukin 1β (IL-1β), interleukin 2 (IL-2). These substances were evaluated in blood serum by the immune enzyme ELISA method. For the “Вектор Бест” test, Company reagents (Russian Federation) and DIA Source ImmunoAssays (Belgium) kits were used. The study was conducted in the Immunology laboratory of “Nicolae Testemitanu” SMPhU. 

SPSS program (version 20.0) was used for the statistical processing of the data. The comparative and descriptive statistics (Student t-test) was used. Data were presented according to Mean ± Standard Error of Mean formula. The significant threshold for comparisons was set at 5% (p < 0.05).

## Results and discussions

58 patients (men - 19, women - 39), aged between 23 and 70 years (46 ± 8.5 years) were included in the study group, in which the immunological peculiarities of complicated urolithiasis were studied depending on the inflammatory process activity. 

**Fig. 1:**
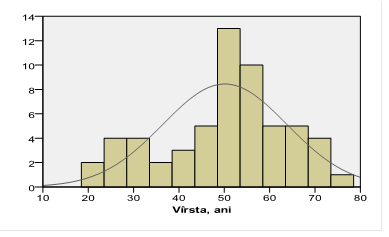
Distribution of patients according to age (years)

The minority of investigated patients were aged 18-30 years (6 persons, 10.3% patients). Most of the investigated patients were aged between 31 and 60 years: 39 people (67.3%). The number of patients aged over 60 years was relatively smaller and was 13 (22.4%) (**Fig. 1**).

Right urinary lithiasis was diagnosed in 33 (56.9%) patients, left urinary lithiasis - 22 (37.9%) patients and bilateral lithiasis - only in 3 (5.2%) cases. Staghorn calculi were found in two (3.4%) patients (**Table 1**), and multiple calculi in 3 (5.2%) patients. 

Various methods of treatment were applied in patients included in the study (**Table 1**). Most patients were treated by pyelolitothomy - 23 (62.2%). The rest of the interventions included: ESWL - 21 (36.2%) patients, ureteroscopy - 12 (20.7%), pyelolitothomy - 25 (43.1%) patients. 

**Table 1:** The studied indices in patients with complicated urolithiasis

Indices	n	%
Pyelonephritis:		
Absent	37	63,8
Latent	9	15,5
Acute	12	20,7
Localization: kidney/ ureter	36/ 22	62,1/ 37,9
Localization: right/ left	33/ 22	56,9/ 37,9
multiple	3	5,2
Coraliform lithiasis	2	3,4
Applied treatment:		
ESWL	21	36,2
Ureteroscopy	12	20,7
Pyelolitothomy	25	43,1

The study immunity results obtained in the study are presented in **Table 2**.

**Table 2:** Cellular and humoral immunity status indices in patients with complicated urolithiasis

Indices (%)	Control group n = 30	Study group n = 58	p
T total	65,3 ± 1,6	53,5 ± 1,2	p < 0,001
T active	27,6 ± 0,5	18,2 ± 0,7	p < 0,001
T morals	27,0 ± 3,0	17,6 ± 0,8	p < 0,001
T suppressors	12,3 ± 1,5	19,2 + 1,4	p < 0,01
T helpers	44,0 ± 2,0	34,5 ± 1,6	p < 0,001
B lymphocytes	14,0 ± 0,7	10,8 ± 0,3	p < 0,001
Ig G, g/ l	12,4 ± 0,3	9,68 ± 0,32	p < 0,001
Ig А, g/ l	2,7 + 0,15	1,2 ± 0,03	p < 0,001
Ig M, g/ l	1,8 ± 0,07	3,96 + 0,02	p < 0,001
iL-1β, pg/ ml	24,6 + 1,5	68,6 ± 1,5	p < 0,001
iL-2, pg/ ml	2,4 + 0,12	3,6 ± 0,36	p < 0,01

As it was shown in **Table 2**, in patients in the active stage of chronic calculus pyelonephritis, a significant decrease of T-lymphocytes compared to the normal indexes (from 65.3 ± 1.6 to 53.5 ± 1.2%, p < 0.001) was detected in the majority from the Т-helper account (from 44 ± 2.0 to 34.5 ± 1.6%, p < 0.001). Increasing the number of Т-suppressor was also significant (from 12.3 ± 1.5 to 19.2 ± 1.4%, p < 0.01). 

The content of B lymphocytes decreased 1.3 times compared to the norm (from 14.0 ± 0.7 to 10.8 ± 0.3%, p < 0.001), IgА - 2.3 times (from 2.7 ± 0.15 to 1.2 ± 0.03 g/ l, p < .001), IgG - 1.3 times (from 12.4 ± 0.3 to 9.68 ± 0 32 g/ l, p < 0.001) with the values increasing in plasma levels Ig М (1.8 + 0.07 to 3.96 ± 0.02 g/ l, p < 0.001). Interleukin-1β obtained indices exceeded the normal values 2.7 times, being statistically significant (from 24.6 + 1.5 to 68.6 ± 1.5, p < 0.001), whereas the values of interleukin-2 - only 1.5 times (from 2.4 ± 0.12 to 3.6 ± 0.36, p < 0.01). 

For this reason, in the obtained results in our study, we noticed the presence of a severe immunodeficiency, with a functional inability of T and B cells, in the patients with complicated urolithiasis, which fundamented the conclusion of prescribing not only antibiotics, but also the effective immunomodulatory therapy for the treatment of chronic calculus pyelonephritis. 

The performed studies enabled us to show the fact that renal lithiasis results in significant changes of cellular, humoral and nonspecific immunity, but the quasi constant increase of the concentration of biologically active proinflammatory substances in the blood serum reflected a state of chronic inflammation, these changes being more expressed at the cellular and humoral immunity level. Assessing the immune status in these patients allowed the appreciation of drug treatment tactics in the pre- and post-surgery period. Disorders of the immune status were followed by the significant reduction of cellular immunity with decreased levels of IgG and IgA and IgM serum growth values increase, interleukins 1β and 2 which required additionally to basic therapy with antibiotics and an effective contemporary immunomodulatory therapy.

**Conclusions**

**1.** Renal lithiasis results in significant changes in the organism’s immune system, these changes being better expressed at the cellular and humoral immunity level.

**2.** The immune status assessment in these patients allows the appreciation of drug treatment tactics in the pre- and post-surgery period.
